# Draft Genome Sequences of Two *Vibrionaceae* Species, *Vibrio ponticus* C121 and *Photobacterium aphoticum* C119, Isolated as Coral Reef Microbiota

**DOI:** 10.1128/genomeA.01095-14

**Published:** 2014-10-30

**Authors:** Nurhidayu Al-saari, Pedro Milet Meirelles, Sayaka Mino, Wataru Suda, Kenshiro Oshima, Masahira Hattori, Moriya Ohkuma, Fabiano L. Thompson, Bruno Gomez-Gil, Toko Sawabe, Tomoo Sawabe

**Affiliations:** a Faculty of Fisheries Sciences, Hokkaido University, Hakodate, Japan; b Laboratory of Microbiology, Institute of Biology and SAGE-COPPE, Federal University of Rio de Janeiro (UFRJ), Rio de Janeiro, Brazil; c Center for Omics and Bioinformatics, Graduate School of Frontier Sciences, University of Tokyo, Kashiwa, Japan; d Microbe Division/Japan Collection of Microorganisms, RIKEN BioResource Center, Ibaraki, Japan; e A.C. Unidad Mazatlán, CIAD, Mazatlán, México; f Department of Food and Nutrition, Hakodate Junior College, Hakodate, Japan

## Abstract

Here, the draft genome sequences of two *Vibrionaceae*, *Vibrio ponticus* C121 and *Photobacterium aphoticum* C119, which were isolated from the coral reef vicinity in Okinawa, Japan, are reported. The genome provides further insight into the genomic plasticity, biocomplexity, and ecophysiology, including pathogenicity and evolution, of these genera.

## GENOME ANNOUNCEMENT

The family *Vibrionaceae* encompasses diverse metabolically versatile marine bacteria that are associated with many significant events in the marine ecosystem, including fish/shellfish pathogenicity (1), mutual symbiosis with marine organisms (2, 3), and mediation of the nutrient cycle in the marine food web (4, 5). They are ubiquitous in marine environments and are characterized by high genomic plasticity (6). These dynamics have made the differentiation of sister species difficult (7). Interestingly, such characteristics have also nominated *Vibrionaceae* species as a great test model for genomic taxonomy (7).

To date, the family *Vibrionaceae* accounts for 142 validly described species and the number is growing continuously (http://www.bacterio.net/-candidatus.html). However, the rapid expansion of the family has also demanded more reliable, reproducible, and informative schemes to define a new species that can be attained using microbial genomic taxonomy. While the type strains of *V. ponticus* (8) and *P. aphoticum* (9) were described in 2004 and 2011, respectively, no genome sequence has been reported since then. Our study reports the first draft genome sequences of *V. ponticus* C121 and *P. aphoticum* C119, which were isolated from water samples taken near the coral reef vicinity of Iriomote-Ishigaki National Park, Okinawa, Japan. *V. ponticus* strain was isolated not only from seawater and mussels but also diseased sea bream (8) and Japanese sea bass (10). *P. aphoticum* was isolated from seawater (9). The species might be found closed as a clade of *P. rosenbergii* on the basis of multilocus sequence analysis (11). The data presented here is aimed to extend the current database of *Vibrionaceae* and subsequently assist further elucidation in the genomics, pathogenicity, and evolution of this group of bacteria.

The genome sequences of *V. ponticus* C121 and *P. aphoticum* C119 were sequenced with the Ion PGM System (Life Technologies, Carlsbad, CA) and assembled using Newbler version 2.8. The annotation and genome analysis were performed by RAST (Rapid Annotation Subsystem Technology) (12). The size of the draft genome of *V. ponticus* C121 is 4,658,121 bp, comprising 98 contigs with a G+C content of 45.2%. On the other hand, the size of the draft genome of *P. aphoticum* C119 is 5,412,298 bp, comprising 56 contigs with a G+C content of 49.7%. The redundancies of both were 71 and 26, and *N*_50_ contig lengths were 128,889 bp and 217,077 bp, respectively. The numbers of putative coding sequences (CDS) were 5,654 for *V. ponticus* C121 and 6,976 for *P. aphoticum* C119; of rRNA sequences were 3 and 6; and of tRNA sequences were 78 and 61, respectively.

These strains have been deposited in the Japan Collection of Microorganisms as JCM 19238 (C121) and JCM 19237 (C119), respectively.

### Nucleotide sequence accession numbers.

The genome data have been deposited at DDBJ/EMBL/GenBank under the accession numbers BBMI01000001 to BBMI01000098 for *Vibrio ponticus* C121 and BBMN01000001 to BBMN01000056 for *Photobacterium aphoticum* C119.

## References

[1] Austin B Zhang X-H. 2006. *Vibrio harveyi*: a significant pathogen of marine vertebrates and invertebrates. Lett. Appl. Microbiol. 43:119–124. 10.1111/j.1472-765X.2006.01989.x 16869892

[2] Ruby EG Urbanowski M Campbell J Dunn A Faini M Gunsalus R Lostroh P Lupp C McCann J Millikan D Schaefer A Stabb E Stevens A Visick K Whistler C Greenberg EP. 2005. Complete genome sequence of *Vibrio fischeri*: a symbiotic bacterium with pathogenic congeners. Proc. Natl. Acad. Sci. U. S. A. 102:3004–3009. 10.1073/pnas.0409900102 15703294 PMC549501

[3] Ruby EG. 1996. Lessons from a cooperative, bacterial-animal association: the *Vibrio fischeri-Euprymna scolopes* light organ symbiosis. Annu. Rev. Microbiol. 50:591–624. 10.1146/annurev.micro.50.1.591 8905092

[4] Takemura AF Chien DM Polz MF. 2014. Associations and dynamics of *Vibrionaceae* in the environment, from the genus to the population level. Front Microbiol. 5:38. 10.3389/fmicb.2014.00038 24575082 PMC3920100

[5] Thompson JR Polz MF. 2006. Dynamics of *Vibrio* populations and their role in environmental nutrient cycling, p 190–203. *In* Thompson FL Austin B Swings J (ed), The biology of vibrios. ASM Press, Washington, DC

[6] Thompson FL Klose KE AVIB Group. 2006. Vibrio2005: the first international conference on the biology of vibrios. J. Bacteriol. 188:4592–4596. 10.1128/JB.00141-06 16788166 PMC1483023

[7] Thompson CC Vicente ACP Souza RC Vasconcelos ATR Vesth T Alves N Ussery DW Iida T Thompson FL. 2009. Genomic taxonomy of vibrios. BMC Evol. Biol. 9:258. 10.1186/1471-2148-9-258 19860885 PMC2777879

[8] Macián MC Garay E Grimont PAD Pujalte MJ. 2004. *Vibrio ponticus* sp. nov., a neighbour of *V. fluvialis*-*V. furnissii* clade, isolated from gilthead sea bream, mussels and seawater. Syst. Appl. Microbiol. 27:535–540. 10.1078/0723202041748127 15490554

[9] Lucena T Ruvira MA Pascual J Garay E Macián MC Arahal DR Pujalte MJ. 2011. *Photobacterium aphoticum* sp. nov., isolated from coastal water. Int. J. Syst. Evol. Microbiol. 61:1579–1584. 10.1099/ijs.0.025171-0 20675441

[10] Xie ZY Hu CQ Zhang LP Chen C Ren CH Shen Q. 2007. Identification and pathogenicity of *Vibrio ponticus* affecting cultured Japanese sea bass, *Lateolabrax japonicus* (Cuvier in Cuvier and Valenciennes). Lett. Appl. Microbiol. 45:62–67. 10.1111/j.1472-765X.2007.02141.x 17594462

[11] Sawabe T Ogura Y Matsumura Y Feng G Amin AR Mino S Nakagawa S Sawabe T Kumar R Fukui Y Satomi M Matsushima R Thompson FL Gomez-Gil B Christen R Maruyama F Kurokawa K Hayashi T. 2013. Updating the *Vibrio* clades defined by multilocus sequence phylogeny: proposal of eight new clades, and the description of *Vibrio tritonius* sp. nov. Front Microbiol. 4:414. 10.3389/fmicb.2013.00414 24409173 PMC3873509

[12] Aziz RK Bartels D Best AA DeJongh M Disz T Edwards RA Formsma K Gerdes S Glass EM Kubal M Meyer F Olsen GJ Olson R Osterman AL Overbeek RA McNeil LK Paarmann D Paczian T Parrello B Pusch GD Reich C Stevens R Vassieva O Vonstein V Wilke A Zagnitko O. 2008. The RAST server: rapid annotations using subsystems technology. BMC Genomics 9:75. 10.1186/1471-2164-9-75 18261238 PMC2265698

